# Effect of He's* Santong* Needling Method on Dysphagia after Stroke: A Study Protocol for a Prospective Randomized Controlled Pilot Trial

**DOI:** 10.1155/2018/6126410

**Published:** 2018-08-14

**Authors:** Luopeng Zhao, Lu Liu, Claire Shuiqing Zhang, Lin Zeng, Jingxia Zhao, Linpeng Wang, Xianghong Jing, Kelun Wang, Bin Li

**Affiliations:** ^1^Acupuncture and Moxibustion Department, Beijing Hospital of Traditional Chinese Medicine Affiliated to Capital Medical University, Beijing Key Laboratory of Acupuncture Neuromodulation, Beijing, China; ^2^Beijing Key Laboratory of Clinic and Basic Research with Traditional Chinese Medicine on Psoriasis, Beijing Institute of Traditional Chinese Medicine, Beijing, China; ^3^Institute of Acupuncture and Moxibustion, China Academy of Chinese Medical Sciences, Beijing, China; ^4^School of Health and Biomedical Sciences, RMIT University, Melbourne, Victoria, Australia; ^5^Research Centre of Clinical Epidemiology, Peking University Third Hospital, Beijing, China; ^6^Center for Sensory-Motor Interaction (SMI), Department of Health Science & Technology, Aalborg University, Aalborg, Denmark

## Abstract

**Background:**

Dysphagia is a common complication of stroke, affecting up to 78% of stroke patients. The existence of dysphagia after stroke has been associated with an increased risk for pulmonary complications and even mortality. Previous studies have shown that acupuncture could be potential therapeutic method for treatment of dysphagia after stroke. A prospective randomized controlled pilot trial is designed to evaluate the effect of He's* Santong* needling method on dysphagia after stroke.

**Methods and Design:**

Sixty eligible participants will be recruited and randomly assigned into treatment group (He's* Santong* needling method and swallowing rehabilitation training) and control group (swallowing rehabilitation training) in a 1:1 ratio. All treatments will be provided everyday on weekdays with a two-day interval at the weekend, during a total treatment course of four weeks. The Penetration-Aspiration Scale with Fiberoptic Endoscopic Examination of Swallowing will be assessed at baseline and endpoint (Week 4) as primary outcomes. The Saitoh's classification score, Swallowing-Related Quality of Life score, the Modified Mann Assessment of Swallowing Ability score, and Surface Electromyography will be evaluated at baseline and endpoint as secondary outcomes.

**Ethics and Dissemination:**

The trial protocol has been approved by the Research Ethical Committee of Beijing Hospital of Traditional Chinese Medicine Affiliated to Capital Medical University on 9 May 2017 (ethical batch number: 2017BL-013-02). Each participant will be notified regarding the study protocol. Written informed consent will be obtained from each participant.

**Trial Registration:**

ISRCTN registry: ISRCTN68981054; Registered on 25 September 2017.

## 1. Background

Dysphagia after stroke, manifested as difficulty in swallowing, is a common complication affecting between 37% and 78% of stroke patients [1]. Although the majority of patients achieve recovery from dysphagia spontaneously, dysphagia is still present in 11–50% of stroke sufferers at six months after stroke onset. Persistent dysphagia puts them at risk of pneumonia, malnutrition, dehydration, and even death [2]. It has been confirmed that the existence of dysphagia is associated with poor outcome during the subsequent years [3].

Swallowing is considered one of the most complex neuromuscular activities, which involves 26 pairs of muscles, five cranial nerves, and a complex neural network which coordinates swallowing muscles [4]. Sensory input from the oral cavity, pharynx, and larynx to the central pattern generator (CPG) has been shown to affect swallowing initiation, facilitation, and airway protection [5]. Dysphagia after stroke is thought to be due to damage to the swallowing-related cortex and subcortical structures. The recent researches have demonstrated that increased sensory input can drive long-term beneficial neural changes and reorganize brain plasticity of the swallowing cortex [6].

In order to improve the recovery and shorten the recovery time, various therapeutic options exist for dysphagia after stroke, including exercise-based rehabilitation, drugs, surgical rehabilitation, noninvasive brain stimulation (NBS), and neuromuscular electrical stimulation (NMES) [7]. There is no recommended routine clinical treatment for dysphagia after stroke, although several studies have shown that each approach appears to be promising [2, 7]. Effective alternative therapies are needed to be developed.

Acupuncture is widely utilized in interventions in complications after stroke in the Asia-Pacific region, although the specific mechanism remains to be further explored [8]. Previous studies found that acupuncture could improve the blood supply of vertebrobasilar artery and blood microcirculation in pseudobulbar palsy and regulate the connection of the cortex and the swallowing center of the brainstem to control swallowing reflection and coordinate motor movement of the swallow related muscles [9–11]. It is speculated that functional reconstruction of the neural network which coordinates swallowing-related muscles and nerves will lead to swallowing recovery [5]. According to traditional Chinese medical theory, acupuncture treatment performs function by the regulation of Qi and blood of swallowing-related meridian and collateral. Three meta-analysis researches have indicated that acupuncture may have a beneficial positive effect on dysphagia after stroke [12–14].

In clinical practice, acupuncture is often combined with other therapeutic stimuli, the so-called combined acupuncture technique, which has been widely used to enhance the acupuncture effect. He's Santong needling method is a combined acupuncture technique, which is often applied to complications after stroke [15]. Compared with ordinary acupuncture, He's Santong needling method has a synergistic effect of three acupuncture techniques: Weitong (normal needling), Wentong (fire needling), and Qiangtong (bloodletting) [16]. The procedures of Weitong method are very similar to ordinary acupuncture, which is well known in the western world. However, most western physicians are not familiar with Wentong (fire needling) and Qiangtong (bloodletting) [17]. Fire needling is an acupuncture technique involving the swift pricking of acupuncture points or certain areas of body with a red-hot needle made of manganese and tungsten alloy [18]. This technique provides the patients with both mechanical stimulation and thermal stimulation, so it is regarded as a combination of acupuncture with thermal stimulation which warm up the meridian to harmonize the Qi and blood with the heat of the fire needling [19]. Some reports have shown that the therapeutic effect of fire needle is superior to ordinary acupuncture on several diseases including acute cerebral infarction [20–22]. It is believed that fire needling has a synergistic effect of heat from the red-hot needle and stimulation on acupuncture points or the certain areas of body in improving blood circulation and eliminating blood stasis [23]. Bloodletting, known as pricking bloodletting method, is a therapeutic acupuncture technique where a small amount of blood from a superficial vein is released with a three-edged needle [18]. It is thought that pricking superficial vein for bloodletting can dredge the meridians and collaterals and promote blood circulation to remove stasis. Therefore, bloodletting has been applied in various types of diseases including stroke for thousands of years in China. Previous study revealed that bloodletting increased the cerebral blood flow in ischemic patients which might improve local microcirculation and prevent neuron apoptosis [24].

Thus, we hypothesize that He's Santong needling method is beneficial to dysphagia after stroke. We design a prospective randomized controlled pilot trial to investigate the effect of He's Santong needling method on dysphagia after stroke.

## 2. Methods

### 2.1. Study Design

**Figure 1:**
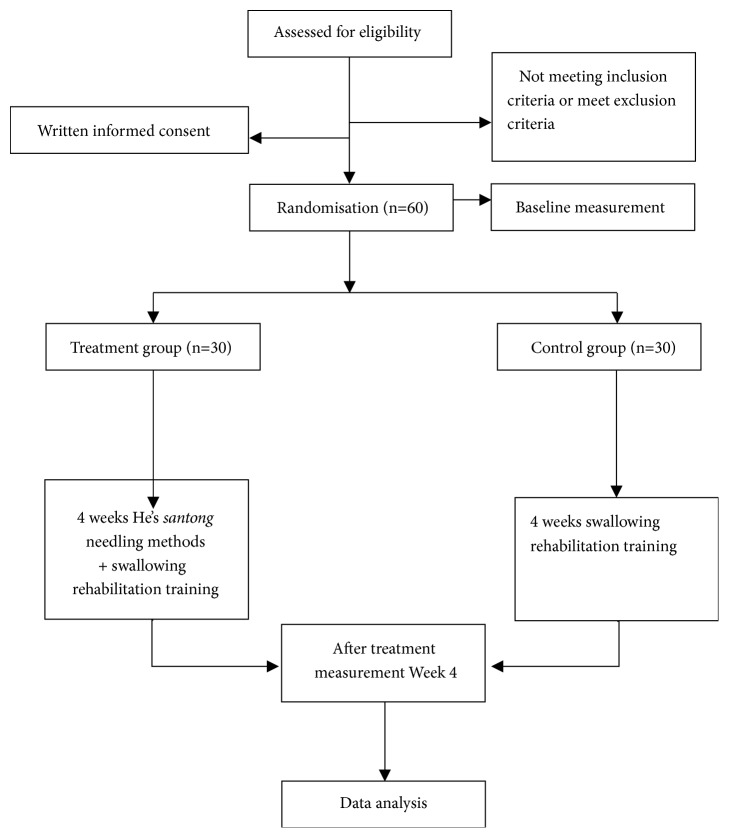
Flowchart of the trial protocol.

A prospective randomized controlled pilot trial is designed to evaluate the effect of He's* Santong* needling method on dysphagia after stroke. Sixty participants with dysphagia after stroke will be randomly assigned to the treatment group (He's* Santong *needling and swallowing rehabilitation training) and control group (swallowing rehabilitation training) at a 1:1 ratio. The schedule of enrolment, interventions, and assessments is summarized in Table 1, and the flow diagram of the study procedure is presented in Figure 1.

### 2.2. Recruitment

Trial participants with dysphagia after stroke are being recruited by clinicians from Acupuncture and Moxibustion wards at the Beijing Hospital of Traditional Chinese Medicine Affiliated to Capital Medical University. Meanwhile, information flyers introducing the details of the trial are being posted at the inpatient and outpatient clinics for greater exposure.

### 2.3. Study Procedure

The treatment is performed 5 days a week for 4 weeks. Once potential participants show interest in this trial, they will be invited to attend an eligibility assessment in which their eligibility will be assessed by trial investigators; thereafter, eligible participants will be enrolled and randomly assigned to either the treatment group or the control group and given a 4-week treatment.

### 2.4. Participants

#### 2.4.1. Inclusion Criteria

Participants who meet all of the following requirements will be allowed for enrolment: (1) aged between 18 and 80 years old, (2) diagnosed as having ischemic stroke which confirmed by a CT or MRI scan, (3) identified as clinical dysphagia using Kubota water swallowing test (level 3, 4, or 5), (4) the symptoms of dysphagia lasting over two weeks to three months after stroke onset, and (5) absence of cognitive impairment (score of the abbreviated mental test (AMT) >=8).

#### 2.4.2. Exclusion Criteria

Patients will be excluded if they have (1) presence of dysphagia before stroke, (2) progressive neurological disorder, (3) serious psychological disorder, (4) a history of pacemaker implantation, (5) unstable cardiopulmonary status, and (6) pregnancy, lactation, or insufficient contraception.

### 2.5. Randomization and Allocation Concealment

The Research Centre of Clinical Epidemiology, Peking University Third Hospital, will be responsible for random program. A block randomization method (with a block size of four) will be used to generate the random allocation sequence; predetermined computer-made randomization opaque sealed envelope will be used to ensure the allocation concealment. According to the serial number of participant, a numbered envelope contains the group assignment will be opened.

### 2.6. Blinding and Informed Consent

In this study, the participants will be informed that they have a 50% chance of being allocated in either of the two groups: He's* Santong* needling method plus swallowing rehabilitation training in the treatment group and swallowing rehabilitation training in the control group. Hence participants will not be blinded to their group allocation. Furthermore, it is unfeasible to blind the acupuncturist because of the nature of the intervention; they will be required to minimize communication with participants or outcome assessor regarding treatment procedures and responses. However, outcome assessors and statisticians will be blind to allocation throughout the entire trial.

### 2.7. Interventions

The acupuncture points were determined according to records in ancient and modern books and results of previous research on acupuncture treatment for dysphagia after stroke [14]. All participants will go through a standardised interview and will be provided with details of the study. The acupuncturist who deliver treatments for treatment group are registered with the Ministry of Health of the People's Republic of China as Chinese medicine practitioners and have more than 20 years clinical experience. Before the trial begins, all acupuncturists will receive special training regarding the purpose and standard procedure of the trial, treatment strategies, and quality control. The treatment details will be fully documented in accordance with the Standard Protocol Items for Randomized Trials (STRICTA) and good clinical practice guidelines [25].

All participants will receive 4-week therapy for five sessions each week. In the treatment group, participants will receive He's* Santong* needling method and swallowing rehabilitation training. In the control group, participants will be treated with swallowing rehabilitation training.

He's* Santong* needling method has three acupuncture procedures:* Weitong* (normal needling),* Wentong* (fire needling), and* Qiangtong* (bloodletting). The* Weitong *procedure will be received five sessions every week, and the* Wentong* and* Qiangtong* procedure will be given two sessions per week on weekdays.

The swallowing rehabilitation training consists of indirect behavioral exercises (e.g., appropriate dietary modification) and direct behavioral exercises (e.g., effortful swallowing and supraglottic swallow technique) according to the Chinese expert consensus for stroke rehabilitation evaluation and treatment, under the direction of rehabilitation practitioners [26]. The 30-minute swallowing rehabilitation session will be carried out by the same rehabilitation practitioner for each participant.

Both groups will receive secondary prevention of ischemic stroke including antiplatelet, antihypertensive, and hypolipidemic therapy according to Chinese Guidelines for Diagnosis and Treatment of Acute Ischemic Stroke [27].

#### 2.7.1. The Acupuncture Points Used in He's Santong Needling Method

**Figure 2:**
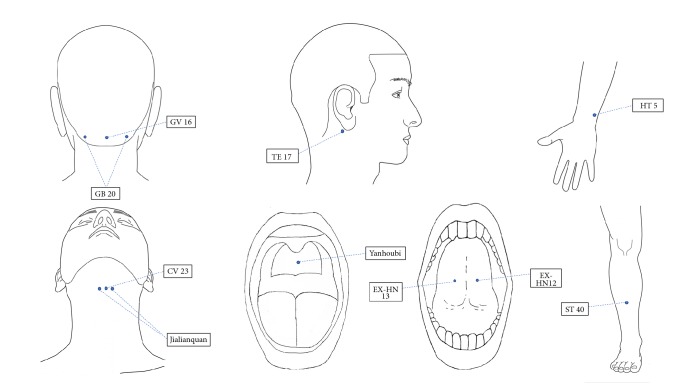
Acupuncture points used in He's* Santong* needling method.

*(1) Weitong (Normal Needling)*. The points in the* Weitong *procedure (needle retention for 30 minutes) are bilateral GB20 (Fengchi), GV16 (Fengfu), TE17 (Yifeng), CV23 (Lianquan), Jialianquan, ST40 (Fenglong), and HT5 (Tongli) (see Figure 2).

All needles will be inserted 10 to 15 mm in depth and manually manipulated with rotation methods to produce a characteristic sensation known as De Qi (feeling of needle sensation that refers to the tenseness around the needle felt by the practitioner and numbness, distension, soreness, and heaviness around the point felt by the participant).

*(2) Wentong (Fire Needling)*. The points in the* Wentong *procedure are bilateral GB20 (Fengchi) and CV23 (Lianquan) (see Figure 2).

The fire needle used in this study is made of manganese and tungsten alloy which will maintain hardness at high temperatures. When the fire needle has been burnt red-hot over a spirited lamp, the acupuncturist will insert the red-hot needle into GB20 and CV23 to a depth of 10mm and then withdraw swiftly without needle retention. The needle hole will be pressed with a sterilized dry cotton-ball [28].

*(3) Qiangtong (Bloodletting)*. The points in the* Qiangtong *procedure are EX-HN12 (Jinjin), EX-HN13 (Yuye), and Yanhoubi (see Figure 2)

Before bloodletting on EX-HN12 and EX-HN13, the acupuncturist will raise the tongue with a spatula and stab the sublingual veins to release 1-2 drops of blood with a three-edged needle after strict disinfection. When performing on the Yanhoubi, the acupuncturist will keep the participant's mouth open with a retainer and press the tongue with a spatula and release 1-2 drops of blood from the pharynx posterior wall with a three-edged needle after strict disinfection [28].

### 2.8. Outcome Measurement

#### 2.8.1. Primary Outcome

*Penetration-Aspiration Scale (PAS) with Fiberoptic Endoscopic Examination of Swallowing (FEES)*. Participants will receive the Fiberoptic Endoscopic examination of Swallowing (FEES) through the fiberoptic endoscopy. They are requested to swallow stained solid food (1 × 1 × 0.5 cm), semi-liquid food (5 ml), and flow food (5 ml and 10ml) in order. If aspiration occurs, the assessment will be terminated. Aspiration will be defined as the entry of stained food into the airway below the levels of the true vocal cords. Silent aspiration will be defined as aspiration occurring in the absence of cough or gag reflex [29]. PAS is an 8-point scale to quantify penetration and aspiration severity that ranges over four stages of oral intake: level 1 (no laryngeal penetration), levels 2~5 (laryngeal penetration), levels 6~7 (tracheal aspiration), and level 8 (silent aspiration) [30]. The combination of FEES and the PAS has been shown to provide reliable and valid assessments [31]. Two experienced clinician assessors will provide a PAS score independently. If they are in disagreement, the expert assessor will confirm a PAS score via consensus.

#### 2.8.2. Secondary Outcomes

*Saitoh's Classification of Dysphagia*. Saitoh's classification divides the swallowing function into 7 levels: (1) saliva aspirator, (2) food aspirator, (3) water aspirator, (4) chance aspirator, (5) oral problems, (6) minimal problems, and (7) normal. A lower score means more severe swallowing disorder [32].

*Swallowing-Related Quality of Life (SWAL-QOL)*. The scale consists of 44 entries, including appetite, food choice, eating time, symptom frequency, psychological burden, fear, language communication, mental health, fatigue, sleep, and social interaction, summaries all aspects of the quality of life of patients with dysphagia. Each entry has 5 different levels ranging from 1 to 5; a lower score represents more severe dysphagia [33].

*The Modified Mann Assessment of Swallowing Ability (MMASA)*. MMASA is a 100-point scale screening tool for identifying eating and swallowing disorders, which includes 12 items from the Mann Assessment of Swallowing Ability (MASA). The 12 items of the MMASA were alertness, cooperation, respiration, expressive dysphasia, auditory comprehension, dysarthria, saliva, tongue movement, tongue strength, gag, voluntary cough, and palate movements. A lower score means more severe swallowing disorder [34].

*Surface Electromyography (sEMG)*. Activity of submental muscles will be evaluated through sEMG, using a Flexcomp Biomonitoring System. sEMG activity will be detected in the anterior belly of the digastric with a pair of electrodes. Participants will adopt a sitting upright position during the sEMG recording. Two electrodes will be placed on the anterior belly of the digastric muscle parallel to longitudinal axis of the muscle, with the reference electrode placed 3 cm apart from the recording electrode. The peak maximum amplitude will be recorded during 2 ml water swallowing. The recordings will be repeated three times and the mean value will be recorded [35].

Abovementioned outcome measurements will be repeatedly assessed at Week 0 and Week 4. Detailed time points of each outcome assessment are provided in Table 1.

### 2.9. Safety Assessment

All adverse events (AEs) during the treatment will be recorded on AE case report in detail, such as time of appearance, intensity of AEs, and possible causes. The investigators will collect information about AEs and assess whether the AEs are associated with the techniques of acupuncture treatment. Participants will be interviewed about any abnormal reactions or feelings. Participants encountering with mild or moderate AEs will be treated according to their symptoms. Severe AEs will be reported to the Research Ethics Committee, which will provide medical advice to the research team within 48 hours, and the Research Ethics Committee will determine whether a termination of the trial is required.

### 2.10. Data Management

All researchers will receive formal training regarding the data collection and management. The data will be recorded into computer by two independent researchers. If any differences are noted, corrections will be made based on the original records in CRFs. All paper documents will be saved in a locked filing cabinet, while electronic documents will be stored in a password-protected computer which will be accessible only to the principal investigators.

### 2.11. Sample Size

This pilot study is to evaluate the effect of He's* Santong* needling method on dysphagia after stroke. However, no previous study was found on the effect of acupuncture on the PAS with FEES. A sample size of 30 per group was regarded as a reasonable minimum recommendation for a pilot study [36]. Therefore, we plan to recruit 60 participants.

### 2.12. Statistical Analysis

Statistical analysis will be performed by statisticians that are blind to allocation of group and intervention process. SPSS 22.0 software (International Business Machines Corporation) will be used for statistical analysis. The intention-to-treat population will be the main set for all efficacy analysis. This population will consist of all randomly allocate participants regardless of the type of treatment received. The per-protocol set will be utilized for sensitivity and consistency analysis to compare the results from the intention-to-treat set. All statistical tests will be two-sided, and p<0.05 is considered statistically significant.

One sample of the Kolmogorov-Smirnov test will be used to test the normal distribution of continuous variables. Continuous variables will be shown as means± Standard deviations (SDs) if they are normally distributed or as medians with IQRs if they are not normally distributed. If the measurement data have normal distribution, independent two-sample* t*-tests will be used for comparisons among the groups, while paired* t*-tests will be used for within-group comparisons. If the measurement data are not normally distributed, the Mann–Whitney* U* test will be used for comparisons among the groups, while Wilcoxon signed-ranks test will be used for within-group comparisons.

### 2.13. Ethics and Dissemination

The trial protocol has been approved by the Research Ethical Committee of Beijing Hospital of Traditional Chinese Medicine Affiliated to Capital Medical University on 9 May 2017 (ethical batch number: 2017BL-013-02). This trial was registered at ISRCTN (ISRCTN68981054). Each participant will be notified regarding the study protocol. Written informed consent will be obtained from each participant.

## 3. Discussion

Evidence accumulating from clinical trials has indicated that acupuncture is effective for dysphagia after stroke [14]. In clinical practice, combined acupuncture technique has been widely used to enhance the acupuncture effect. However, the combined acupuncture technique studies are still lacking. Therefore, we design this pilot study to evaluate the effectiveness of He's* Santong* needling method for dysphagia after stroke. The advantages of the trial design are shown in the following two aspects.

### 3.1. Comprehensive Outcome Measures

Instrumental evaluation of dysphagia mainly includes videofluoroscopy swallowing study (VFSS) and FEES. Considering the feasibility, the PAS with FEES is to be selected as the primary outcome. Combined with PAS, FEES is demonstrated as having a high level of agreement with VFSS [31]. Furthermore, VFSS cannot be used at the bedside frequently for follow-up tests because of the necessary exposure to radiation [37]. Compared with VFSS, the FEES is a more appropriate assessment tool for dysphagia assessment after stroke, especially for our study which concerns oral and pharyngeal phase of swallowing [38, 39]. For stroke patients, a bedside test will ensure patient's comfort and compliance.

Saitoh's classification of dysphagia, SWAL-QOL, MMASA, and sEMG will be used as secondary outcome. Saitoh's classification of dysphagia is an ordinary tool to assess the swallowing function of a patient, which has been utilized to evaluate swallowing function in dysphagia patients [32]. SWAL-QOL is a valid, effective, and sensitive tool to assess quality of life of people with dysphagia, which has been used in many studies involved dysphagia management [33]. The MMASA is a useful screening tool for evaluating eating and swallowing functions, which showed relatively high sensitivity and specificity for detecting aspiration [34]. The sEMG is a noninvasive assessment to monitor electromyographic signal of submental muscles, so it is a safe and repeatable method for stroke patients to evaluate strength of swallowing muscles [35].

### 3.2. Combined Acupuncture Technique

It has been demonstrated that ordinary acupuncture is an effective and safe treatment for dysphagia after stroke [40]. Compared with ordinary acupuncture, He's* Santong* needling method (*Weitong* (normal needling),* Wentong* (fire needling), and* Qiangtong* (bloodletting)) is a combined acupuncture technique, which is often applied to complications after stroke. For effective aspect, He's* Santong* needling method has the potential to increase therapeutic efficacy for dysphagia after stroke, since it combines three monotherapies. For safety aspect, fire needling and bloodletting have been widely used in China for thousands of years [23]. A set of national standard* standardised manipulations of acupuncture and moxibustion* has been established to ensure the safety of acupuncture techniques including bloodletting and fire needling (GB/T 21709.12-2009 and GB/T 21709.4-2008) [28].

It should be noted that our study has certain methodological limitation. Blinding is a crucial measure to assure the quality of the trial. As a limitation, it is difficult to implement blinding in participants because we will adopt a special acupuncture method. In order to overcome this problem, we will minimize the interaction between the participants and acupuncturists. In addition, all outcome assessors and statisticians will be kept blind throughout the whole process.

To our knowledge, this trial is the first attempt to investigate the efficacy of He's* Santong* needling method on dysphagia after stroke. The results of this trial may develop the clinical treatment of dysphagia. However, the biological mechanism of this therapy requires further investigation.

## Figures and Tables

**Table 1:** Schedule of enrolment, interventions, and assessments.

**Baseline**		**Treatment**			
		**week 1**	**week 2**	**week 3**	**week 4**
**Enrolment**	****				
**Eligibility screen**	**×**				
**Informed consent**	**×**				
**Allocation**	**×**				
**Interventions**					
**Treatment group**		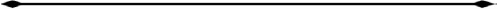			
**Control group**	****	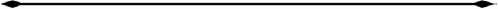			
**Assessments**	****				
**PAS with FEES**	**×**				**×**
**Saitoh's classification**	**×**				**×**
**SWAL-QOL**	**×**				**×**
**MMASA**	**×**				**×**
**sEMG**	**×**				**×**
**Adverse events**	****	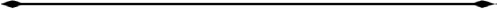			

## Data Availability

The data used to support the findings of this study are available from the corresponding author upon request.

## Abbreviations

AEs: Adverse events
AMT: The abbreviated mental test
CPG: Central pattern generator
FEES: Fiberoptic Endoscopic examination of Swallowing
MMASA: The Modified Mann Assessment of Swallowing Ability
NA: Nucleus ambiguous
NBS: Noninvasive brain stimulation
NMES: Neuromuscular electrical stimulation
NTS: Nucleus tractus solitaries
PAS: Penetration-Aspiration Scale
sEMG: Surface Electromyography
SWAL-QOL: Swallowing-Related Quality of Life
VFSS: Videofluoroscopy swallowing study.

## Additional Points

*Trial Status.* Participants are currently being recruited.

## Supplementary Materials

Supplementary 1 SPIRIT checklist document.

Supplementary 2 Ethical approval document for this trial.

Supplementary 3 Beijing Traditional Chinese Medicine Administration Funding Support, the first funding support document.

Supplementary 4 Beijing Municipal Hospital Administration Funding Support, the second funding support document.

Supplementary 5 National Basic Research Program of China Funding Support, the third funding support document.

Supplementary 6 Table for locations and manipulation of acupuncture points used in He's* Santong* needling method.
