# Supplementary material for: Effect of He's Santong Needling Method on Dysphagia after Stroke: A Study Protocol for a Prospective Randomized Controlled Pilot Trial
Source: Evid Based Complement Alternat Med. 2018 Aug 14;2018:6126410. doi: 10.1155/2018/6126410 (PMC6112255; doi:10.1155/2018/6126410)
Supplement: Supplementary 1 — SPIRIT checklist document. [file 6126410.f1.docx]

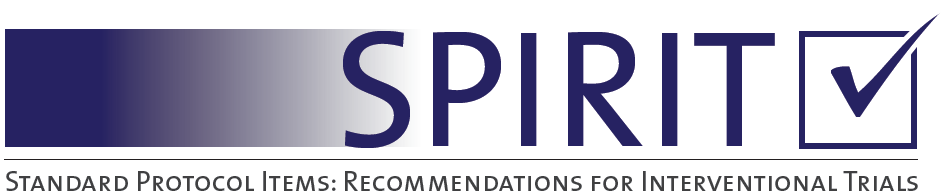


SPIRIT 2013 Checklist: Recommended items to address in a clinical trial protocol and related documents*

| Section/item | Item No | Description | Addressed on page number |
| --- | --- | --- | --- |
| **Administrative information** | | |  |
| Title | 1 | Effect of He’s santong needling method on dysphagia after stroke: a study protocol for a prospective randomised controlled pilot trial | ______1_______ |
| Trial registration | 2a | Trial registration number: ISRCTN68981054 | ______2______ |
|  | 2b |  | _____________ |
| Protocol version | 3 | 2018-04-16 Version1.0 | _____________ |
| Funding | 4 | Beijing Traditional Chinese Medicine Administration, Beijing Municipal Hospital Administration (grant number PZ2017030) and National Basic Research Program of China (grant number 2014CB543203) | ______15______ |
| Roles and responsibilities | 5a | BL, K-LW and X-HJ conceived the study. L-PZ and LL drafted the protocol. C-SZ and J-XZ participated in the design of the study and contributed to the refinement of the protocol. LZ was responsible for the statistical design of the study. L-PW provided clinical advice and made critical revisions. BL is a principal investigator of the study and has the final responsibility for the decision to submit this manuscript for publication. All authors approved the final manuscript. | ______15______ |
|  | 5b | Beijing Traditional Chinese Medicine Administration (Telephone number: 0086 010 83970677) | _____________ |
|  | 5c | Beijing Traditional Chinese Medicine Administration is the sponsor and will not affect the result of this trial. | _____________ |
|  | 5d | This trial will be monitored by the scientific research department of Beijing Hospital of Traditional Chinese Medicine affiliated to Capital Medical University. | _____________ |
| Introduction |  |  |  |
| Background and rationale | 6a | Dysphagia is a common complication of stroke, affecting up to 78% of stroke patients. The existence of dysphagia after stroke has been associated with an increased risk for pulmonary complications and even mortality. Previous studies have shown that acupuncture could be potential therapeutic method for treatment of dysphagia after stroke. A prospective randomised controlled pilot trial is designed to evaluate the effect of He’s *santong* needling method on dysphagia after stroke.  Acupuncture is widely utilized in interventions in complications after stroke in the Asia-Pacific region, although the specific mechanism remains to be further explored. Previous studies found that acupuncture could improve the blood supply of vertebrobasilar artery and blood microcirculation in pseudobulbar palsy, regulate the connection of the cortex and the swallowing centre of the brainstem to control swallowing reflection and coordinate motor movement of the swallow related muscles. It is speculated that functional reconstruction of the neural network which coordinate swallowing related muscles and nerves will lead to swallowing recovery. According to traditional Chinese medical theory, acupuncture treatment performs function by the regulation of Qi and Blood of swallowing related meridian and collateral. Three meta-analysis researches have indicated that acupuncture may have a beneficial positive effect on dysphagia after stroke. | _______3,4______ |
|  | 6b | In clinical practice, acupuncture often combined with other therapeutic stimuli, so called combined acupuncture technique, which has been widely used to enhance the acupuncture effect. He’s santong needling method is a combined acupuncture technique, which is often applied to complications after stroke. Compared with ordinary acupuncture, He’s santong needling method has a synergistic effect of three acupuncture techniques: Weitong (normal needling), Wentong (fire needling), and Qiangtong (bloodletting). Thus, we hypothesize that He's santong needling method is beneficial to dysphagia after stroke. | ______4______ |
| Objectives | 7 | We design a prospective randomised controlled pilot trial to investigate the effect of He’s santong needling method on dysphagia after stroke. | ______5_____ |
| Trial design | 8 | A prospective randomised controlled pilot trial | ______5_______ |
| Methods: Participants, interventions, and outcomes | | |  |
| Study setting | 9 | A single centre in Beijing Hospital of Traditional Chinese Medicine affiliated to Capital Medical University | ______5_______ |
| Eligibility criteria | 10 | **Inclusion Criteria**  Participants who meet all of the following requirements will be allowed for enrolment: (1) aged between 18 and 80 years old, (2) diagnosed as having ischemic stroke which confirmed by a CT or MRI scan, (3) identified as clinical dysphagia using Kubota water swallowing test (level 3, 4 or 5), (4) the symptoms of dysphagia lasting over two weeks to three months after stroke onset, (5) absence of cognitive impairment (score of the abbreviated mental test (AMT) >7).  **Exclusion Criteria**  Patients will be excluded if they have: (1) presence of dysphagia before stroke, (2) progressive neurological disorder, (3) serious psychological disorder, (4) a history of pacemaker implantation, (5) unstable cardiopulmonary status, (6) pregnancy, lactation, or insufficient contraception. | ______6______ |
| Interventions | 11a | The acupuncture points were determined according to records in ancient and modern books and results of previous research on acupuncture treatment for dysphagia after stroke. All participants will go through a standardised interview and be provided with details of the study. The acupuncturist who deliver treatments for treatment group are registered with the Ministry of Health of the People’s Republic of China as Chinese medicine practitioners, and have more than 20 years clinical experience. Before the trial begins, all acupuncturists will receive special training regarding the purpose and standard procedure of the trial, treatment strategies, and quality control. All participants will receive 4-week therapy for five sessions each week. In the treatment group, participants will receive He’s *santong* needling method and swallowing rehabilitation training. In the control group, participants will be treated with swallowing rehabilitation training. | ______7______ |
|  | 11b | The He’s *santong* needling method has three acupuncture procedures: *Weitong* (normal needling), *Wentong* (fire needling), and *Qiangtong* (bloodletting). The *Weitong* procedure will be received five sessions every week, and the *Wentong* and *Qiangtong* procedure will be given two sessions per week on weekdays.  The swallowing rehabilitation training consists of indirect behavioural exercises (eg, appropriate dietary modification) and direct behavioural exercises (eg, effortful swallowing, supraglottic swallow technique) according to the Chinese expert consensus for stroke rehabilitation evaluation and treatment, under the direction of rehabilitation practitioners. The 30-minute swallowing rehabilitation session will be carried out by the same rehabilitation practitioner for each participant. | _____7______ |
|  | 11c | All participants will receive free treatment | _____________ |
|  | 11d | Both groups will receive secondary prevention of ischemic stroke including antiplatelet, antihypertensive and hypolipidemic therapy according to Chinese Guidelines for Diagnosis and Treatment of Acute Ischemic Stroke. | _____8_______ |
| Outcomes | 12 | Primary outcome  Penetration-Aspiration Scale (PAS) with Fiberoptic Endoscopic examination of Swallowing (FEES)  Secondary Outcomes  Saitoh’s classification of dysphagia, Swallowing-Related Quality of Life (SWAL-QOL), The Modified Mann Assessment of Swallowing Ability (MMASA), Surface Electromyography (sEMG) | _____9,10_____ |
| Participant timeline | 13 | Table 1 | ______20______ |
| Sample size | 14 | This pilot study is to evaluate the effect of He’s *santong* needling method on dysphagia after stroke. However, no previous study was found on the effect of acupuncture on the PAS with FEES. A sample size of 30 per group was regarded as a reasonable minimum recommendation for a pilot study. Therefore, we plan to recruit 60 participants. | ______11______ |
| Recruitment | 15 | Trial participants with dysphagia after stroke are being recruited by clinicians from Acupuncture and Moxibustion wards at the Beijing Hospital of Traditional Chinese Medicine Affiliated to Capital Medical University. Meanwhile, information flyers introducing the details of the trial are being posted at the inpatient and outpatient clinics for greater exposure. | ______5______ |
| **Methods: Assignment of interventions (for controlled trials)** | | |  |
| Allocation: |  |  |  |
| Sequence generation | 16a | The Research Centre of Clinical Epidemiology, Peking University Third Hospital will be responsible for random program. A block randomisation method (with a block size of four) will be used to generate the random allocation sequence; predetermined computer-made randomisation opaque sealed envelope will be used to ensure the allocation concealment. | _____6____ |
| Allocation concealment mechanism | 16b | According to the serial number of participant, a numbered envelope contains the group assignment will be opened. | ______6______ |
| Implementation | 16c | In this study, the participants will be informed that they have a 50% chance of being allocated in either of the two groups: He’s *santong* needling method plus swallowing rehabilitation training in the treatment group and swallowing rehabilitation training in the control group.. | _____6______ |
| Blinding (masking) | 17a | Hence participants will not be blinded to their group allocation. Furthermore, it is unfeasible to blind the acupuncturist because of the nature of the intervention; they will be required to minimize communication with participants or outcome assessor regarding treatment procedures and responses. However, outcome assessors and statisticians will be blind to allocation throughout the entire trial. | ______6,7______ |
|  | 17b |  | _____________ |
| **Methods: Data collection, management, and analysis** | | |  |
| Data collection methods | 18a | All researchers will receive formal training regarding the data collection and management. The data will be recorded into computer by two independent researchers. If any differences are noted, corrections will be made based on the original records in CRFs | ______11_______ |
|  | 18b | Dropouts and withdrawals from the study will be recorded in detail. | _____________ |
| Data management | 19 | All paper documents will be saved in a locked filing cabinet, while electronic documents will be stored in a password-protected computer which will be accessible only to the principal investigators. | _______11______ |
| Statistical methods | 20a | Statistical analysis will be performed by statisticians that are blind to allocation of group and intervention process. SPSS 22.0 software (International Business Machines Corporation) will be used for statistical analysis. The intention-to-treat population will be the main set for all efficacy analysis. This population will consist of all randomly allocate participants regardless of the type of treatment received. The per-protocol set will be utilized for sensitivity and consistency analysis to compare the results from the intention-to-treat set. All statistical tests will be two-sided, and p<0.05 is considered statistically significant. | _____11_____ |
|  | 20b | One sample of the Kolmogorov-Smirnov test will be used to test the normal distribution of continuous variables. Continuous variables will be shown as means± Standard deviations (SDs) if they are normally distributed or as medians with IQRs if they are not normally distributed. If the measurement data have normal distribution, independent two-sample t-tests will be used for comparisons among the groups, while paired t-tests will be used for within-group comparisons. If the measurement data are not normally distributed, the Mann-Whitney U-test will be used for comparisons among the groups, while Wilcoxon signed-ranks test will be used for within-group comparisons.. | ______11____ |
|  | 20c |  | __________ |
| **Methods: Monitoring** | | |  |
| Data monitoring | 21a | This trial will be monitored by the scientific research department of Beijing Hospital of Traditional Chinese Medicine affiliated to Capital Medical University. | _____________ |
|  | 21b |  | _____________ |
| Harms | 22 | All adverse events (AEs) during the treatment will be recorded on AE case report in detail, such as time of appearance, intensity of AEs, and possible causes. The investigators will collect information about AEs and assess whether the AEs are associated with the techniques of acupuncture treatment. Participants will be interviewed about any abnormal reactions or feelings. Participants encountering with mild or moderate AEs will be treated according to their symptoms. Severe AEs will be reported to the Research Ethics Committee, which will provide medical advice to the research team within 48 hours, and the Research Ethics Committee will determine whether a termination of the trial is required. | ______10,11_____ |
| Auditing | 23 | The study will be audited by Beijing Traditional Chinese Medicine Administration every year. | _____________ |
| Ethics and dissemination | | |  |
| Research ethics approval | 24 | The trial protocol has been approved by the Research Ethical Committee of Beijing Hospital of Traditional Chinese Medicine Affiliated to Capital Medical University on 9 May 2017 (ethical batch number: 2017BL-013-02). This trial was registered at ISRCTN (ISRCTN68981054). | ______12_____ |
| Protocol amendments | 25 | If it is nessrary to modify the protocol, we should submit applications to Beijing Municipal Administration of Traditional Chinese Medicine | _____________ |
| Consent or assent | 26a | Each participant will be notified regarding the study protocol. Written informed consent will be obtained from each participant. | ______12______ |
|  | 26b |  | _____________ |
| Confidentiality | 27 | All paper documents will be saved in a locked filing cabinet, while electronic documents will be stored in a password-protected computer which will be accessible only to the principal investigators. | ______11______ |
| Declaration of interests | 28 | The authors declare that they have no competing interests. | ______15_______ |
| Access to data | 29 | All paper documents will be saved in a locked filing cabinet, while electronic documents will be stored in a password-protected computer which will be accessible only to the principal investigators. | ______11_______ |
| Ancillary and post-trial care | 30 |  | _____________ |
| Dissemination policy | 31a | The results will be published after the study. | _____________ |
|  | 31b | Researcers in this trial will have authorship eligibility. | _____________ |
|  | 31c | The results will be published. | _____________ |
| Appendices |  |  |  |
| Informed consent materials | 32 | Written informed consent will be obtained from all participants. | _____________ |
| Biological specimens | 33 |  | _____________ |

*It is strongly recommended that this checklist be read in conjunction with the SPIRIT 2013 Explanation & Elaboration for important clarification on the items. Amendments to the protocol should be tracked and dated. The SPIRIT checklist is copyrighted by the SPIRIT Group under the Creative Commons “[Attribution-NonCommercial-NoDerivs 3.0 Unported](http://www.creativecommons.org/licenses/by-nc-nd/3.0/)” license
