# Supplementary material for: Effect of He's Santong Needling Method on Dysphagia after Stroke: A Study Protocol for a Prospective Randomized Controlled Pilot Trial
Source: Evid Based Complement Alternat Med. 2018 Aug 14;2018:6126410. doi: 10.1155/2018/6126410 (PMC6112255; doi:10.1155/2018/6126410)
Supplement: Supplementary 2 — Ethical approval document for this trial. [file 6126410.f2.docx]

伦理审查批件 ER.03.03-V1.04

Ethical Approval Document

首都医科大学附属北京中医医院医学伦理委员会

Research Ethical Committee of Beijing Hospital of Traditional Chinese Medicine

Affiliated to Capital Medical University

伦理审查批件 审批号：2017BL-013-02

Ethical Approval Document Approval number:2017BL-013-02

| 项目名称  Research name | 贺氏三通法治疗中风后吞咽障碍  Effect of He’s santong needling method on dysphagia after stroke | | | | | |
| --- | --- | --- | --- | --- | --- | --- |
| 项目来源  Funding | 北京市中医药管理局  Beijing Municipal Administration of traditional Chinese Medicine | | | | | |
| 临床研究负责单位  Research Institution | 首都医科大学附属北京中医医院  Beijing Hospital of Traditional Chinese Medicine affiliated to Capital Medical University | | | | | |
| 本院主要研究者  Investigator | 李彬  Bin Li | | | | | |
| 审查类别  Approval category | 复审审查  Review | | | 审查方式  Approval procedure | | 快递审查  Express Review |
| 审查日期  Review date | 2017年5月9日  **9, May ,2017** | | | | | |
| 审查机构  Review Institution | 首都医科大学附属北京中医医院  Beijing Hospital of Traditional Chinese Medicine affiliated to Capital Medical University | | | | | |
| 批准文件  Approval files | 研究方案：版本号：20170501，版本日期：2017年05月01日；  Assignment for technical design (Version 20170501; Date: 20170501).  知情同意书：版本号：20170501，版本日期：2017年05月01日；  Informed consent form (Version 20170501; Date: 20170501).  病例报告表：版本号：20170501，版本日期：2017年05月01日。  Case report form (Version 20170501; Date: 20170501). | | | | | |
| 审查结果  Review comment | 同意1票  Approval 1 | | 不同意0票  Disapproval 0 | | 做必要的修正后同意0票  Modification required prior to approval 0 | |
|  | 做必要的修正后重审0票  Modification required and re-submitted for review 0 | | | | 终止或暂停试验0票  Terminate or suspend its prior approval 0 | |
| 审查意见  Review recommendation | | | | | | |
|  | | 根据卫生部《涉及人的生物医学研究伦理审查办法》（2007），国家中医药管理局《中医药临床研究伦理审查管理规范》（2010），SFDA《药物临床试验伦理审查工作指导原则》（2010）、《药物临床试验质量管理规范》（2003）、《中药品种保护指导原则》（2009）、《医疗器械临床试验规定》（2004）、以及WMA《赫尔辛基宣言》（2008）和CIOMS《人体生物医学研究国际伦理指南》（2002）的伦理原则，经本医学伦理委员会审查，同意按照所批准的临床研究方案、知情同意书、招募材料等开展本项试验/研究。  According to the World Medical Association Declaration of Helsinki Ethical Principles for Medical Research Involving Human Subject, International Ethical Guidelines for Biomedical Research Involving Human Subjects by the Council for International Organization of Medical Sciences and several Chinese ethical guidelines,the assignment for technical design, informed consent form and case report form were approved by the Research Ethical Committee of Beijing Hospital of Traditional Chinese Medicine affiliated to Capital Medical University.  请遵循GCP规定和本伦理委员会批准的方案开展临床研究。  Please carry out the research  该项目进行中如发生下列情况，须及时书面报告本伦理委员会：  The investigator should report to the Ethical Committee if the following occurs:   1. 对临床方案、知情同意书等的任何修改；   1) Amendment of research protocol and informed consent form.   1. 更换主要研究者；   2) Change of major investigator.   1. 发生严重不良事件；   3) Serious adverse events.   1. 出现任何可能显著影响试验进行或增加受试者危险的情况；   4)Occurrence of significant influence to the research or increasing harm to participants   1. 出现违反方案情况；   5) Deviation of research protocol   1. 暂停或提前终止临床研究。   6)Research suspension or termination  本伦理委员会将对该项目跟踪审查，请申请人/申办方按照伦理委员会规定的年度或定期跟踪审查频率，在截止日期前1个月提交研究进展报告。  The research ethical committee will track and check the research process according the specified year or frequency, and the research progress report should be submitted a month before the deadline.  该项目完成后，请向本伦理委员会提交结题报告。  Please submit research conclusion report to the research ethical committee  如该项目在批件有限期内未能启动临床研究，本批件作废，需要重新提交伦理审查申请。  If the project can not start within the effective period, this document will be invalid and need resubmitting related documents. | | | | |
| 年度/定期跟踪审查频率  Frequency of continuing review | | 12个月  12monthes  请于2018年04月08日前提交研究进展报告。  Please submit research progress report before 9/11/2016 | | | | |
| 批件有效期  Period of validity | | 2017年05月09日-2018年05月08日  From 10/12/2015 to 9/12/2016 | | | | |
| 联系人与联系电话  Responsible person and telephone | | 王晶 010-64011516  Jing Wang 010-64011516 | | | | |
| 伦理委员会主任/副主任签字  Research Ethical Committee Director/ | | 信 彬  Bin Xin | | | | |
| 首都医科大学附属北京中医医院医学伦理委员会  Research Ethical Committee of Beijing Hospital of Traditional Chinese Medicine  Affiliated to Capital Medical University | | | | | | |
| 日期：2017年05月09日  Date：20170509 | | | | | | |
