# Supplementary material for: Effect of He's Santong Needling Method on Dysphagia after Stroke: A Study Protocol for a Prospective Randomized Controlled Pilot Trial
Source: Evid Based Complement Alternat Med. 2018 Aug 14;2018:6126410. doi: 10.1155/2018/6126410 (PMC6112255; doi:10.1155/2018/6126410)
Supplement: Supplementary 3 — Beijing Traditional Chinese Medicine Administration Funding Support, the first funding support document. [file 6126410.f3.docx]

**北京地区中医康复服务能力**

**与技术平台规范化建设项目方案**

**Standardized construction project of rehabilitation service capability and technology platform of Traditional Chinese Medicine in Beijing**

**贺氏三通法治疗中风后吞咽困难**

**Treatment of dysphagia after stroke with He's santong needling method**

**（版本号：3.0、版本日期：20170501）**

**(**Edition:3.0, Edition date:20170501**)**

**负 责 人：李 彬**

**Chief Investigator: Bin Li**

**联系电话：010-52176910**

**Telephone: 86-010-52176910**

**承担部门：首都医科大学附属北京中医医院针灸科**

**Research Institution: Acupuncture and Moxibustion Department, Beijing Hospital of Traditional Chinese Medicine Affiliated to Capital Medical University**

**资助部门：北京市中医管理局**

**Funding: Beijing Traditional Chinese Medicine Administration**

**2016年9月1日**

**1 September, 2016**
