# Supplementary material for: Effect of He's Santong Needling Method on Dysphagia after Stroke: A Study Protocol for a Prospective Randomized Controlled Pilot Trial
Source: Evid Based Complement Alternat Med. 2018 Aug 14;2018:6126410. doi: 10.1155/2018/6126410 (PMC6112255; doi:10.1155/2018/6126410)
Supplement: Supplementary 4 — Beijing Municipal Hospital Administration Funding Support, the second funding support document. [file 6126410.f4.docx]

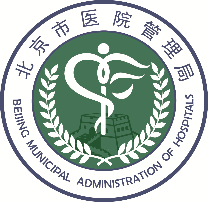
附件2

P Z 2 0 1 7 0 3 0

Grant number: PZ2017030

Additional file 2

**北京市属医院**

**科研培育计划项目申报书**

**Beijing Municipal Hospital**

**Scientific research and training program project**

**（2017版）**

**（version 2017）**

项目名称： 新型金针针具的研制及六寸金针治疗良性甲状腺结节的临床疗效观察

Program name: Development of a New Gold Needle and Clinical Observation of Six-inch Gold Needle in Treating Benign Thyroid Nodules

二级学科： 针灸学

Subtitle: Acupuncture and Moxibustion

三级学科：

所属专业： 针灸

Title: Acupuncture

项目类别：西医（ ）中医（√）

Project Category: Western Medicine () Chinese Medicine (√)

项目领域：医疗（ ）护理（ ）药械（√）医技（ ）应用基础（ ）

其他（ ）

Project Area: Medical () Nursing () Medical equipment (√) Medical Technology () Application Basics () Other ()

申报单位： 首都医科大学附属北京中医医院

Institution: Beijing Hospital of Traditional Chinese Medicine Affiliated to Capital Medical University

申 报 人： 李 彬

Applicant: Bin Li

联系电话： 010-52176055

Telephone: 86-010-52176055

填表日期： 2016年5月28日

[Date :](https://www.baidu.com/link?url=G-55fW4hl6jxAPX2k5PLvxhucnhtuelqibtq1swPqzf_gJYiKZO-Dd6SO53ASV5jSh3972uVskMGsQwAH7iO8wHLkdHlbCFHsc_d9czEQEVylj2Yx3UkQyaXKDhgedYEQHlkaTW1kwO2CnizH8k39q&wd=&eqid=cd6f48f000054db1000000035a07f086) May 28, 2016

**北京市医院管理局**

Beijing Municipal Hospital Authority
