# Supplementary material for: Effect of He's Santong Needling Method on Dysphagia after Stroke: A Study Protocol for a Prospective Randomized Controlled Pilot Trial
Source: Evid Based Complement Alternat Med. 2018 Aug 14;2018:6126410. doi: 10.1155/2018/6126410 (PMC6112255; doi:10.1155/2018/6126410)
Supplement: Supplementary 5 — National Basic Research Program of China Funding Support, the third funding support document. [file 6126410.f5.doc]

**国家重点基础研究发展计划**

National Basic Research Program of China

1. **计划）**

（973 Plan）

**课题任务书**

Program

项目名称：**腧穴配伍效应规律及神经生物学机制研究**

Program name: Study on the effect regularity and neurobiological mechanism of acupoints compatibility

课题编号：**2014CB543203**

Grant number: 2014CB543203

课题名称：**腧穴配伍效应差异的神经生物学机制研究**

Research name: Study on neurobiological mechanism of differences in acupoints compatibility

**中华人民共和国科学技术部制**

Ministry of science and technology of the People's Republic of China

**二〇一三年八月**

August 2013

1
