# Supplementary material for: Effect of He's Santong Needling Method on Dysphagia after Stroke: A Study Protocol for a Prospective Randomized Controlled Pilot Trial
Source: Evid Based Complement Alternat Med. 2018 Aug 14;2018:6126410. doi: 10.1155/2018/6126410 (PMC6112255; doi:10.1155/2018/6126410)
Supplement: Supplementary 6 — Table for locations and manipulation of acupuncture points used in He's Santong needling method. [file 6126410.f6.docx]

| Points | Location^18^ | Manipulation |
| --- | --- | --- |
| GB20 (Fengchi) | ‘In the anterior region of the neck, inferior to the occipital bone, in the depression between the origins of sternocleidomastoid and the trapezius muscles.’ | *Weitong*: Insert at a depth of  1-1.5 cun in the direction to  the throat.  *Wentong*: When the needle is burnt red, insert at a depth of 0.5-1 cun and withdraw swiftly without needle retention in the direction to apex nasi. |
| GV16 (Fengfu) | ‘In the posterior region of the neck, directly inferior to the external occipital protuberance, in the depression between the trapezius muscles.’ | *Weitong*: Insert at a depth of  1-1.5 cun in the direction to  the throat underjaw. The tip of the needle should not be raised so as not to injure the medulla oblongata through the foramen magnum. |
| TE17 (Yifeng) | ‘In the anterior region of the neck, posterior to the ear lobe, in the depression anterior to the inferior end of the mastoid process.’ | *Weitong*: Insert at a depth of 1.5-2 cun in the direction to throat. |
| CV23 (Lianquan) | ‘In the anterior region of the neck, superior to superior border to thyroid cartilage, in the depression superior to the hyoid bone, on the anterior median line.’ | *Weitong*: Insert obliquely at a depth of 1.5-2 cun in the direction to root of tongue.  Wentong: When the needle burns red, insert and withdraw swiftly without needle retention at a depth of 0.5-1 cun, in the direction to root of tongue. |
| Jialianquan | jialianquan is one cun lateral to CV23. | Similar to CV23 |
| ST40 (Fenglong) | ‘On the anterolateral aspect of the leg, lateral border of the tibialis anterior muscle, 8 B-cun superior to the prominence of the lateral malleolus.’ | *Weitong*: Insert at a depth of  1.5-2 cun. |
| EX-HN13 (Yuye) | ‘On the underside of the tongue, on the lingual vein to the right of the frenulum.’ | *Qiangtong*: Raise the tongue with a spatula, a three-edged needle is used to release 1-2 drops of blood under strict sterile conditions. |
| EX-HN12 (Jinjin) | ‘On the underside of the tongue, on the lingual vein to the left of the frenulum.’ | Similar to EX-HN13 |
| Yanhoubi | Pharynx posterior wall | *Qiangtong*: Open the opening with a retainer and press the tongue with a spatula, a three-edged needle is used to prick blood. |
